# Chronic pain and masculine identity: life-world interviews with men at a South African Pain Clinic

**DOI:** 10.1080/17482631.2021.1970303

**Published:** 2021-08-26

**Authors:** David Blackbeard, Colleen Aldous

**Affiliations:** a Department of Clinical Psychology, Grey’s Hospital, Pietermaritzburg, South Africa; b Department of Psychiatry, College of Health Sciences, Nelson R. Mandela School of Clinical Medicine, University of KwaZulu-Natal, Durban, South Africa; c College of Health Sciences, Nelson R. Mandela School of Clinical Medicine, University of KwaZulu-Natal, Durban, South Africa

**Keywords:** Chronic pain, Dialogical Self Theory, Gender Relations Theory, interview dialogues, life-world interviews, lived experience, masculinity, masculine identity, patient experience

## Abstract

**Purpose:**

The purpose was to investigate experiences of men who were living with chronic pain in relation to masculine identity and their experiences of treatment at a Chronic Pain Clinic in South Africa.

**Methods:**

A purposive sample of 14 male patients from an outpatient Chronic Pain Clinic participated in the study in 2019. Qualitative inquiry followed a life-world dialogical interview approach. Respondent validation interviews further engaged participant perspectives. Team data analysis, thematic network diagrams and tabulations were used for analysis of the interview data. .

**Results:**

The respondents described multiple challenges of the journey to chronic pain, living with chronic pain, experiences of treatment contexts, ways of coping and the experience of living with chronic pain in relation to masculinity. Three typologies were identified: (1) aligning with hegemonic ideals, (2) a yielding masculinity or (3) an adjusted masculine identity. The findings revealed how masculine identity was positioned by the perceptions of others, interpersonally and within the individual.

**Conclusions:**

Healthcare practitioners and public health can be responsive to the gendered context of living with difficult and long-term pain conditions. Treatment should be supportive and inclusive..

## Introduction

Our study concerned the lived experience of male patients attending a chronic pain clinic at a South African public sector hospital. The concept of hegemonic masculinity from critical masculinity studies (Connell & Messerschmidt, 2005; R. W. Connell, 1995) and concepts of embodied self and identity positioning from Dialogical Self Theory (Hermans, 2015; Hermans & Kempen, 1993) were used to analyse constructions of masculine identity within the experiential context of living with chronic non-cancer pain (hereafter referred to as chronic pain), including experiences of the treatment context. Individual life-world dialogical interviews (Kvale & Brinkmann, 2015; Tangaard, 2009) were used to engage participants using a semi-structured interview schedule (Table 1). Our interest was in the dynamic positioning of masculine identity against perceived ideal constructions of masculinity by men living with chronic pain.

**Table I.** Semi-structured interview items

Could you tell me about your experience of chronic pain? Could you tell me about your experiences of treatment for your chronic pain? Could you tell me about your experience of being a man with chronic pain? Could you tell me about your experience of masculinity and having chronic pain, such as expectations and perceptions related to being a man? How has living with chronic pain changed your life? What has been less or more difficult about being a man living with chronic pain? Do you have anything you wish to add or any questions?

The gendered contexts of medical conditions, patient experience and treatment outcomes have received increasing attention by researchers in healthcare, treatment and rehabilitation. An emerging focus in patient experience research is the interaction of living with long-term health conditions with identity, including gender identity (Sallinen, Mengshoel, & Solbrække, 2019) and gendered embodiment (Bernardes & Lima, 2010). ‘Sex difference’ research in health and illness has tended to overlook psychological, social and cultural construction of sex and gender, non-binary constructions and gender fluidity (Bernardes et al., 2008). More recently, researchers in gender and health have engaged theory from gender studies, including critical masculinity studies to better understand the gendered experience of individuals with health conditions (Bernardes et al., 2008). Groundbreaking research has been done on the gendered experience of individuals with chronic pain. These researchers have used qualitative methods and theoretical perspectives from gender studies (Ahlsen et al., 2014, 2012; Rovner et al., 2017). In one such study, a contrast was observed between rational and defended masculine identity, in which feelings of vulnerability or loss were minimized or controlled, against a meaningful and adjusted masculine identity in which feelings of vulnerability and loss were made permissible (Ahlsen et al., 2012).

In recent decades, a theoretical mainstream has developed around masculinity as a social construction. These theories replaced the notions of essential masculinity based in biological difference or fixed social roles. Among other theories, Raewyn Connell’s theory of multiple masculinities (R. W. Connell, 1995) developed as a perspective on the many faces of masculinity in relation to gendered power. Central to Connell’s theory was the concept of hegemonic masculinity, the culturally desirable or influential ideal for masculinity that maintains patterns of gender relations in social institutions, cultural contexts and social groups (Connell & Messerschmidt, 2005). Connell’s earlier work in masculinities emphasized the multiple positions of masculinity formed in relation to a culturally ideal masculinity, such as subjugation, marginalization, complicity or identification (R. W. Connell, 1995). R.W. Connell (2002) formulated that these multiple positions functioned to maintain patterns of gendered power relations, such as the “costs and benefits” of patriarchal systems. In patriarchal systems, the ‘benefits’ of partriachy for men, also called a “patriarchal dividend” comes with considerable “costs” to groups and individual men (Connell & Messerschmidt, 2005). The cost of group and individual positioning against an ideal masculinity is that these ideals can be premised on control, fear of vulnerability, subjugation, exclusion and self-reliance.

Work on the psychology of masculine identity has also placed hegemonic masculinity within interpersonal and intrapsychic domains (Blackbeard, 2018). This was in keeping with Connell’s initial case study approach for analysing masculinity of individuals within social or occupational groups (R. W. Connell, 1995). To this end, researchers in South Africa integrated the hegemonic masculinity concept with a system or bridging theory for understanding self and identity, H.J.M. Hermans’ Dialogical Self Theory (Blackbeard, 2018; Hermans, 2015). This integration with DST, maps the positioning of masculine identity within the internal and external relations self and other within culture and society, and is expanded upon further in this paper (Blackbeard, 2018).

Research in masculinity and health conditions has highlighted the links of masculinity with health-related attitudes and behaviours (Keogh, 2015). Masculinity based in responsibility and relationality (value given to interpersonal and social relationships) is positively associated with health protective behaviours, whereas masculinity based on toughness and self-reliance is associated with delayed help-seeking, help avoidance, risky lifestyle habits, and medical non-adherence (Fleming & Agnew-Brune, 2015). Researchers have also identified how men living with long-term medical conditions adjust or reposition masculine identity (Flurey et al., 2018). Men living with rheumatoid arthritis, for example, realigned themselves with ideal masculinity by retaining or renegotiating ideals or by embracing alternative ideals (Flurey et al., 2018). Similar patterns of adjusted identities were noted in research on men with fibromyalgia (Sallinen, Mengshoel, & Solbrække, 2019), osteoporosis (Nielsen et al., 2011) and men’s experiences of bariatric surgery (Groven et al., 2015). Bernardes and Lima (2010) noted that an individual with chronic pain faced a disconnection between the lived reality of living with pain and gender ideals, having to then adjust to a new sense of self as a gendered person. A potential need in such research has been to theorize how masculinity, particularly in its embodiment, is readjusted or maintained in the context of health challenges. We suggest in this paper that a consideration of hegemonic masculinity from the perspective of Dialogical Self Theory (DST) provides a perspective for formulating how embodied masculine identity changes and is changed in the context of a health condition such as chronic pain.

Before proceeding with the theoretical perspective, the context of chronic pain is overviewed to further inform the rationale for the study. It is widely recognized that chronic pain is characterized by persistent pain symptoms beyond the primary stimulus or injury (Baliki & Apkarian, 2015). The relationship between the primary source of pain and its subsequent chronicity, persistence, and development is complex, with substantial evidence for chronic pain neural patterns emerging in the patient with chronic pain (Baliki & Apkarian, 2015). The experience of chronic pain often has social and psychological components, unlike most acute pain, which is more directly linked to a primary physiological source/s of the pain (Oraison & Kennedy, 2019). It is also well accepted that chronic is challenging for individuals, partners, families and health systems. Multiple studies confirm that chronic pain is associated with significant psychosocial challenges such as reduced quality of life, mental health difficulties such as depression and suicide risk, emotional distress, social isolation, occupational difficulties and maladaptive coping (Baliki & Apkarian, 2015; Dutta et al., 2013; Hooley et al., 2014). Attendees at chronic pain clinics are diverse, with differences in both the pathophysiology of pain and demographics such as employment status and ethnicity; studies in African and non-African contexts have shown that chronic pain services are attended more often by women than men, and comprise proportionally more middle and older age groups (May et al., 2018; Rovner et al., 2017; Tidman et al., 2017). For this reason, research on the experience of chronic pain patients can relevantly inform effective biopsychosocial interventions and treatments targeted to the needs of this diverse patient population. Differences have been noted in how men and women present with pain and the characteristics of the pain experience with constructs such as ‘pain acceptance’ or ‘kinesiophobia (Rovner et al., 2017). From such research, it can be considered that chronic pain is multisystemic, and has a multiplicity of challenges experienced by individuals with chronic pain. The diverse context of chronic pain also highlights the need for research on the experience of under-researched groups, such as older men and minorities (Colvin, 2019).

Masculinity is embodied through acts and capacities such as physical activity, physical functionality, bodily changes, strength and endurance (Groven et al., 2015). Bodily experiences can be more or less consciously involved in articulating a masculine identity (Groven et al., 2015). Researchers of men’s experience of chronic pain have described how men with chronic pain develop stories of their “bodily self” and identity in relation to their health, sometimes reformulating self and identity or enriching the self-narrative with themes of restitution and acceptance (Ahlsen et al., 2014). In the re-storying of their experience, men with chronic pain and other long-term health challenges appear to find possibilities for reformulating or readjusting their masculine identities, finding new “positions” as men with limitations for embodying an ideal “normal” masculinity, and adjusting their sense of social value (Groven et al., 2015).

In motivating for this research on masculinity and men’s experience of living with chronic pain, the conceptual complexity of gender was considered, noting the multidimensionality of gender, the embodiment of identity, diversity and fluidity . How men with chronic pain positioned a masculine self and identity against perceived ideals of masculinity was noted to be relevant, noting the many challenges and limitations of living with chronic pain and the likely difficulty in matching previous expectations for masculinity as a performative and embodied identity. This question was relevant because it would potentially unlock how men with chronic pain could be supported in adjusting to life with chronic pain, especially in relation to their meanings of masculinity, self and identity. To inform the conceptualization of this study, an integrated theoretical approach was developed, bridging Connell’s concepts of multiple masculinities and hegemonic masculinity (Connell & Messerschmidt, 2005) with Dialogical Self Theory (DST) (Hermans et al., 2017), which will be explained further.

## The South African context of masculinity

Masculinity studies in the South Africa (SA) have shed light on the positive qualities and challenges of masculinity in a local, global and diverse context (Morrell, 2007). As a lower middle-income country, SA presents a varied landscape of contrasts and disparities among poor and affluent; disadvantaged and privileged; rural, peri-urban and urban; cultures, ethnicities, languages and generations. Masculinity has different meanings in South Africa, and is strongly intersected with differences of social identities, culture, ethnicity and language, and the context of the post-apartheid constitutional democracy, as noted by local masculinity scholars (Morrell, 2007; Morrell et al., 2012). Public health and social interventions with men in South Africa has been focused on various issues, the pressing needs of halting and reducing gender-based violence, men’s access, uptake and participation in health programmes and the deeply rooted challenges of poverty and unemployment (Jewkes et al., 2015). Nurturing positive non-violent masculinities has been an ongoing task, taken up by both the non-governmental organizations (NGOs) and in public programmes (Jewkes et al., 2015). Racialized stereotypes of black and white masculinities have also been problematized and challenged by local scholars, who note that black masculinity is often represented as violent and dangerous (Langa et al., 2020). Moolman (2017) highlights that masculinity in SA is fluid with discourses of rights and gender equity gaining currency among South African men, with a reconstituting of what it means to be a father or a spouse, bolstered by public health interventions and social programmes. Masculinity in SA is therefore a fluid and flexible domain, drawing on modern and traditional influence or the dialogue between these influences (Moolman, 2017).

## Theoretical framework

The concept of hegemonic masculinity developed within critical masculinity studies especially in the work of R.W. Connell (1995); R.W. Connell (2002). Hegemonic masculinity proved useful in conceptualizing the location, enactment and expression of masculinity as a social identity or cultural ideal (Morrell et al., 2012). Hegemonic masculinity can function as a model or standard, identified with implicitly or overtly, or as a comparison or aspiration by individuals or groups of men in society (Colvin, 2019). In further developments, the contextuality of hegemonic masculinity was framed as a set of performative ideals and practices within shifting hierarchies of masculinity (Connell & Messerschmidt, 2005). This idea of masculinity as contextual, performative and embedded in practices also has implications for transforming masculinity towards positive and gender-equal configurations (Jewkes et al., 2015), and the concept of the hegemonic masculinity links constructions of masculinity with gender power relations (Connell & Messerschmidt, 2005).

Hegemonic masculinity can be applied with a “structural focus”, such as larger scale analyses of power and institutions (Connell, 2002); however, it can be well utilized within more personal and interpersonal domains, for understanding the articulation and performance of identities at individual and group levels, and for an analysis of masculine identity (Blackbeard, 2018). Archer (2001) highlighted that masculine identity is produced through positioning of self and other, intersectional with other identity positions. In previous research, the Dialogical Self Theory (DST) of H.J.M. Hermans was found to be a useful bridging theory of self and identity to conceptualize masculine identity in relation to hegemonic masculinity (Blackbeard, 2018; Hermans, 2015). Self and identity are constructed through patterns of multiple dynamic I-positions of varying centrality or dominance with “fields of tension” at the interface of self and society (Hermans et al., 2017).

In seeking to conceptualize masculine identity within a chronic pain context, the concepts of the body and embodiment were crucial, considering the corporeal, the morphological and the symbolic aspects of embodiment. From a perspective of corporeal feminism, the body and its materiality can comprise both a cultural-linguistic entity and a biological presence (Sandberg, 2011). This conception of embodiment matched DST as framework for conceptualizing dynamic and intersectional positioning of an embodied masculine self and identity. In elaborating upon DST, Hermans and Hermans-Konopka (2010) emphasize that voiced I-positions are singular or plural, embodied, dynamic, located, emotionally valenced and in relations of influence with other self- and other-positions, both internal or external to the individual. In terms of embodiment, this implies that self-positions have a meaningful morphology and corporeality. Self-positioning occurs through both the external and material domains of the self, in the physical and social environment but also in relation to the internal representations of the individual in relation to self and others (Hermans & Hermans-Konopka, 2010). In DST, the self is considered from the perspective of the other but also in terms of what is internalized by the self, or the “other-in-self” (Hermans, 2015, p. 2).

Our study was therefore informed by a perspective that masculine identity reflected a process of positioning an embodied self and other in relation to hegemonic as an influential or ideal I-position, actualized in dynamic and contextualized ways and in relation to other voiced positions (Hermans et al., 2017). So for example, in a particular context of time and space, an individual might actualize an embodied position of identification, repudiation or resistance in relation to hegemonic masculinity. In another context of time and space (real, virtual or imagined), the same individual might actualize a different position in relation to hegemonic masculinity or from various perspectives. The same individual might position masculine identity differently in various contexts, such as at a social event, at home with a partner or spouse, or in a virtual or digital environment and in relation to other embodied I-positions such as “I-as-a-father”, “I-as-a-husband” or “I-as-a-patient” or in dialogue among such positions. From this perspective, the positioning of a masculine self and identity can also be constrained or facilitated within the circumstances and influences on the individual or group, for example, being unemployed might limit options for positioning against an perceived ideal that a man should be economically productive. The notion of positioning is helpful for identifying linkages and intersections of self and identity and the social, discursive or material contexts, for example, “I-as-an-older-black-South-African-man” or “I-as-a-provider-for-my-family” or “I-as-a-husband-and-father”.

## Materials and methods

A qualitative approach was most appropriate for exploring men’s experiences of living with chronic pain in relation to masculine identity within their everyday contexts, including the treatment context. Qualitative methodology is most appropriate for investigating experiences and meanings of experiences, and can reveal psychosocial processes more effectively than quantitative approaches (Silverman, 2001) The semi-structured life-world interview based on Kvale and Brinkman (2015) was the primary source of information, with a focus on the respondents as active meaning-making subjects interactive with and influenced by social discourses . The interview approach also included developments by Tanggaard (2009) who had extended upon semi-structured life-world interviewing with the inclusion of dialogue as essential for the co-production of interview meanings. The semi-structured interview schedule was developed from the research question which in turn had been inspired by studies from elsewhere around the experience of men living with chronic pain (Ahlsen et al., 2014; Flurey et al., 2018; Groven et al., 2015). The interview approach was aimed at generating experiential knowledge through the interactional context of interviewer and interviewee—facilitating a meaningful description of the life-world, both social and personal, as contained and co-produced within the interview dialogue (Kvale & Brinkman, 2015; Tanggaard, 2009). The process of developing cogent findings was informed by an active, reflexive dialogical process of engagement by the researching team with the participants throughout the data collection and data analysis processes (Varpio et al., 2017).

### Ethics

Study approval was granted by the University of KwaZulu-Natal Biomedical Research Ethics Committee (BE226/18) and site approval was gained from Grey’s Hospital and KwaZulu-Natal Department of Health (KZ_201810_018). This study was conducted in accordance with the principles of good clinical practice and research ethics as set out in the Declaration of Helsinki (General Assembly of the World Medical Association, 2014). The interviewing team consisted of four trained postgraduate students who conducted interviews in English or Zulu languages, according to the preference of the respondent. Interviewer training, a consultative consent process and debriefing sessions were included and contingencies for risk management were in place, for example, referral of participants in distress for appropriate psychosocial services. The ethical aspiration for the study was that a contextual understanding of patient experiences in relation to masculinity would contribute to effective recognition, assessment, care and treatment for persons with chronic pain as stated in the Declaration of Montreal, specifically access to pain management without discrimination, acknowledgement of pain and relevant information, and access to pain assessment and treatment by adequately trained health care professionals (International Pain Summit of the International Association for the Study of Pain, 2011).

### Participants

The participants in this single-site study were all recruited from chronic pain clinic at a public hospital which offered tertiary level services in KwaZulu-Natal Province, South Africa. A purposive maximum variation sampling strategy was used. Fourteen patients were recruited into the study from a list of the 97 male patients who had attended the clinic in the preceding six month. Seventeen prospective participants were contacted telephonically, of which fourteen patients were recruited and three declined. Participants then attended an informational meeting with the interviewer, and an information sheet and consent form was used for an informed consent process.

There were no transgender patients known to the clinic team but accommodation was made in the research protocol to recruit transgender men or gender-fluid individuals should there be any potential participants. Participants with intellectual disabilities were also not excluded from recruitment into the study but there were no potential participants with intellectual disabilities known to the clinic team. The participants were all self-identified male attendees at the Pain Clinic with an age range of 37 years to 71 years (mean age 57.4 years). Ten of the participants were between the ages of 50 and 69 years.

Demographic and health information was recorded on an interview documentation sheet in a pre-interview meeting with each of the participants. This facilitated an overview of the respondent characteristics as a background for the qualitative inquiry. Participant characteristics are tabulated in the Appendix. The sample was racially and linguistically diverse, with two categories of employment status (unemployed or pensioner) with most having previously worked in technical skilled or semi-skilled occupations. Social class was not explicitly identified, but most were previously from low or middle-income occupations, and were currently could be described as living with low income or in extreme poverty, as all were receiving social grants or pensions. Most of the men were married or had been married.

The diversity of the sample approximated the diverse demographics of the chronic pain clinic from which the sample was drawn. Seven of the interviewees chose to be interviewed in the Zulu language and seven of the interviewees were interviewed in English. Seven of the men had previously had back surgery. Four of the men had a history of motor vehicle accident associated with their pain conditions, and five had a history of occupational injury and one had developed chronic pain after being violently assaulted. There was a wide range in years living with chronic pain and years of receiving treatment for chronic pain. All fourteen men received analgesic medication from the Pain Clinic, and five had previously or were currently receiving psychiatric medication, three had received clinical psychology interventions and nine had recently or previously received interventional treatment (such as caudal blocks), Most had previously or were currently receiving individual physiotherapy.

### Interviews

Interviewing took place over 3 months (07/06/2019 to 17/09/2019) with 14 audio-recorded life-world interviews and seven follow-up interviews completed by the participants with one of four trained interviewers. An interview documentation sheet was used to record the information on the interview (date, time, interviewer), demographic information, medical history and treatments received.

The interview was introduced to the potential participants as “a study that involves interviewing men from the Pain Clinic about their experiences as men living with chronic pain” (from the information sheet). A semi-structured interview guide with open-ended questions was devised to engage the men around each respondent’s experience of chronic pain, treatment, and the experience as a chronic pain in relation to masculinity. A post-interview de-briefing meeting then took place between the interviewer and primary investigator. The interviewers then transcribed and (when the interview had been conducted in Zulu) translated the audio-recorded interviews. Team meetings of the interviewers and primary investigator took place for team debriefing, sharing of insights and reflections on the interview process followed by a team seminar for initial thematic analysis of the transcripts, following other qualitative research projects (Blackbeard & Lindegger, 2015; Groven et al., 2015).

Seven follow-up respondent validation interviews took place in an imbricated manner following the primary interviews. For each respondent validation interview, an individual schedule of open-ended statements was devised based upon the themes identified from the first interview. This allowed for the respondents to verify or disconfirm the understandings that had been generated through the first interview. The standard of the study was therefore enhanced by triangulation, the combination of the data from the interview documentation sheet, field notes, debriefing processes and team discussion, and the transcribed initial and respondent validation interviews.

### Analysis

Transcripts were shared and discussed in team meetings, facilitating a consensual process of identifying tentative themes that were then mapped out using a thematic network diagram, following Attride-Stirling (2001). This process built upon the debriefing meetings held after each interview in which first impressions of the interview was dialogued and documented by interviewer and investigator. Team-based analysis can be useful especially at the familiarization phases of analysis in providing a space for multiple perspectives to arrive at an overall consensual view of the data (Lavik et al., 2018). The team-based analysis then continued with a data analysis seminar in which the investigators jointly read and organized transcript material, categorizing chunks of text according to the semi-structured interview items. These team-based approaches were in keeping with similar procedures used in other qualitative studies such as Groven et al. (2015) and Lavik et al. (2018). This process facilitated more comparison across interviews grouped according to the question items, allowing comparisons to be more easily elicited.

The team process was followed by detailed reading and re-reading of the transcripts by the primary investigators, in which themes were coded and adjusted inductively for the most cogent structure of basic themes, organizing themes and global themes (Attride-Stirling, 2001; Nelson et al., 2018). Utilizing Microsoft Word ® review functions, mark ups, comments and tracking changes, the tabulated three-tier analysis was then anchored with illustrative examples from the text as a summative document. In this process, basic themes and organizing themes were further refined for cogency through re-organizing and merging or removing duplications. The basic themes consisted of statements, the simple premises within the data (transcribed interviews) making sense in the context of other basic themes (Attride-Stirling, 2001). Organizing themes were the more abstract clusters of meaning and signification drawn from the basic themes. Global themes were the super-ordinate claims supported by the data, representing the core of the thematic network (Attride-Stirling, 2001).

## Further considerations

It is well-accepted in qualitative research, either interpretive or descriptive, that time and space of the interview is a crucial consideration, as are the potential power dynamics and identities of the interviewer/s (Blackbeard, 2018; Sallinen et al., 2019). The interviews took place in a closed office in the hospital but not at the Pain Clinic itself, and it was known to the participants that the interviewers were postgraduate students and not clinical staff. The setting provided an appropriate distance by being somewhat away from the Pain Clinic but also sufficiently close to the clinical context for the conversation about challenges, treatment experience and living with chronic pain as a man. The interviewers were all black women in their early twenties which appeared to “disarm” the conversation through a difference in gender and generation. This context appeared to free the conversation to some extent, as the interviewers were not perceived to be authority figures or clinicians.

## Results

### Global themes

The analysis produced six global themes that were (1) chronic pain is challenging, (2) associated problems, (3) the journey to chronic pain, (4) treatment experience, (5) masculinity and chronic pain, and (6) coping strategies. The result presented in this article is focused mostly upon the theme of masculinity and chronic pain but with some reference to elements spread across the other themes including sexual function, bodily strength and physical activity, social and work activities, lifestyle adjustments and breadwinner status.

### Masculinity and chronic pain

The interviews with 14 men living with chronic pain offered three positions in relation to masculine identity: (1) “Aligned masculinity”, (2) “Yielding masculinity” and (3) “Adjusted masculinity”. These typologies co-existed in many of the narratives with various emphases and at times were held with ambivalence or tension. This accorded with the idea of dialogical self-positions in space and time, that there can be multiple actualized self-positions held in various relationships to one another within the self-system with tension, varying emphasis or even in contradiction.

### “Aligned masculinity”

The “aligned masculinity” narrative occurred where there was a minimizing or distancing of the impact of the chronic pain, a sense of an unchanged masculine identity, detached from the embodied limitations and discomfort. This was interpreted as attempts to hold onto an embodied identification with hegemonic ideals such as toughness, control and stoicism, exerted upon the self from within and/or externally through social imperatives or pressures to conform to masculinity ideals. This masculine identity position was set apart from the pain, or persistently continued despite the pain, and was found in statements such as this:

Respondent 13: I feel incapable in certain ways like for work and other things but no I do not feel less of a man not … in any way of that sort.

An instrumental sense of control characterized some of these positions, at times held in tension with the need to adapt to changes.

This position appeared to be experienced with a sense of fragility and had an uneasy, brittle quality. A loss of independence and limits to physical activities or strength was also experienced by Respondent 5 with a sense of unresolved ambivalence, as he held on to a hegemonic ideal that a man should carry heavy items.

Respondent 5: … when we go shopping (.) she manages to carry heavy things and that makes me feel bad as a man now (.) you know that is my job (.1) I do it but when she’s with me she will make sure that she does.

From the “aligned masculinity” position, emotions of vulnerability were unacceptable and needed to be controlled. Respondent 14 disallowed having feelings of sadness or discouragement.

Respondent 14: oh (.) being a man with chronic pain (.) it’s something that came into me I can’t (.) ja I can’t be disheartened I can’t be er sad about it …

Instead of allowing feelings of vulnerability, Respondent 14 here focused on a drive to “push forward” and control the pain, aligning with a hegemonic standard of self-reliance, control and stoicism:

Respondent 14: (…) I always push forward (.) but it came a time now that I eventually just can’t do that (.) but I’m not disheartened about it because whenever I pushed I did what I wanted to do (…) it’s there I’ve got to I’ve got to live with it (.1) and control it (.) I’ve got to live with it and control it

Pressure to conform to hegemonic standards despite was not only internal. A 44 year old Zulu man, Respondent 3, experienced pressure to be tough and stoical from the community in which he lived, which was an informal settlement (characterized by extreme poverty):

Respondent 3: (…) sometimes others understand but it’s rare to find people like that (.) most of them say you have try and act strong … say you have try and act strong you are a man while they [community members] aren’t aware of how much pain you’re experiencing.

Respondent 3, who was one of the younger men, also noted that job-seeking was difficult in his situation but that he was perceived to be “lazy”.

Respondent 3: yeah don’t even try to look for it [employment] because I know what will happen (.) and other people just see you as a lazy person

In the journey to chronic pain, there were moments of denial for some of the men, going back to work or previous activities, Respondent 11 tried to go back to playing sport:

Respondent 11: … before the injuries I used to be the best soccer player (.) I tried to go back football after I got injured (.) but I failed …

### “Yielding masculinity”

The “yielding masculinity” narrative an experience of the losses and constraints extending to the loss of parts of oneself, self-worth and identity, including masculine identity. This position appeared to be experienced as a sense of personal failure, loss and a downward movement. The “yielding masculinity” had an unresolved continued identification with hegemonic ideals of autonomy, toughness and a provider role as central to masculine adequacy premised on contestation and winner-loser competition. The experience of loss or multiple losses was emphasized in many of the accounts. These losses included loss of occupation and occupational activities; the loss of income and financial security; the loss of status in the community; the loss of food security; the loss of daily routine and the loss of sporting or other physical activities.

Respondent 11: (…) this thing really destroyed me (…) I used to be a soccer player and do road running (.) now I do not even watch soccer because watching provokes bad memories (.) this thing really destroyed me

The loss of occupation and occupational activities had clearly been very difficult for most of the men.

Respondent 1: (…) you are sitting with loss of your profession of your loss of income

The men expressed various perspectives around the organizing theme of a male “provider role”. This included feeling like a “failed father”, not living up to his own or family’s expectations as a father or for one of the men, not being the “father of the house” as a Zulu man.

Respondent 6: … because I am supposed to play a role at home (.) and be seen as the father of the house now since there are these restrictions (.) I sometimes see myself as not being a complete man

Across a diversity of cultural backgrounds, there were several of the men who had identified with an expectation that a man should provide for the family. Respondent 7 for example, a 52-year-old man who had multiple back surgeries and had lost his employment in a factory, there were feelings of frustration that he was no longer able to be the breadwinner.

Respondent 7: (…) it’s very frustrating especially when you have got a family (.) you know (.) you need to be like a breadwinner and you know you have to be at work and support your family all that keep them going and the children have to go to school (.2) and all that becomes very frustrating

Being no longer gaining an income through work was experienced as challenging as was frustrated feelings of thwarted adequacy in not being able to work at the same standard as before. Respondent 5 talked about his feelings in reaction to his friends who were working.

Respondent 5: I was a worker I was a hard worker I loved working and now you can’t and it hurts you especially when you see your friends that are going to work and coming back from work

Having or losing the capacity to drive a vehicle, a loss of adequate masculinity and being active in the world and working, and its embodiment as mobility and physical efficacy, was here experienced from the “yielding masculinity” position:

Respondent 5:at the end I used to drive I was a driving man (.) I used to drive just to earn a living but that also got (.) I couldn’t do it anymore because if I am saying I am going to Joburg if I get there I can’t get out of the bed the pain has got me

Physiological challenges also included weight loss, bodily weakness and sexual dysfunction, and a sense of changes affecting the whole body and the whole identity of the person. Respondent 4 was a 69-year-old man with multiple pain conditions. He narrated that he had become a “chronic patient”, closely linked to an identity as an older person.

Respondent 4: because becoming chronic patient with all those various sicknesses that’s my part of a story now (.)

He also indicated that “the whole body changes” with the chronic pain and ageing.

Respondent 4: see and if I wasn’t a chronic person I wouldn’t have so much pain (.) andalso old age being old make your bones to become weak so you have a problem see

Another change noted was then limitation of mobility—loss of agility in walking, not being able to walk up steps, a sense of movements being “locked” or “restricted” or not being able to walk “properly” along with a loss of strength or stamina. Respondents noted difficulties in lifting and doing occupational tasks which required “strength” and “energy”. challenging as a man. The sexual dysfunction was a sensitive topic for the men and although at least partly due to the pain condition, physical trauma and the side-effects of medication but was also connected to a sense of decreased worth or even a sense of fear and failure as a man, husband or partner.

The sexual dysfunction associated with the chronic pain and treatment was a considerable difficulty for several of the men, relating to a sense of being an adequate husband or partner.

Respondent 2: (…) I started to be ashamed of myself (…) about what was happening

A loss of independence and income corresponded with increasing reliance on others for several of the men. From a “yielding masculinity” position, this meant failure or inadequacy.

Respondent 6: (…) I was a man who used to work for himself and now that is difficult (…) now I have to depend with the social grant income given by the government and this is not enough as it doesn’t cover everything.

Being reliant on partners and family members was an area mentioned by several of the men, linked to a sense of diminished masculine autonomy and a loss of an autonomous embodiment, also within cultural expectations of male self-reliance.

Respondent 7 (…) it only changed now when you not helpful on your own and (.) you have to depend on them (.) they must support you now

### “Adjusted masculinity”

The “adjusted masculinity” narrative occurred when the men described a sense of being a changed person, of reframing or reiterating ideal of masculinity to fit the constraints and possibilities of living with chronic pain. This reframing of masculinity was experienced with a sense of resolution and positive acceptance. The “adjusted masculinity” position was associated with a sense of renewed purpose, better relationships and a deepening of personal values.

Some of the men had expressed a sense of no longer being a “normal” or “complete” man or felt “inferior” (Respondent 10) because of the pain (yielding masculinity position). An alternative “adjusted masculinity” also appeared in some accounts, associated with restitution and resilience. Respondent 13, for example, felt that even though he felt proud of having endured difficulties:

Respondent 13: no (.) in fact what I have endured (…) I am not happy but I am proud I went through this (…) I feel incapable in certain ways like for work and other things (.) but no (.) I do not feel less of a man (.) not not in any way of that sort

A loss of physical strength was also reframed as a contrast with a perception of character strength although this was always an intrapersonal reality. Respondent 5 depicted these contrasts between external and internal experiences of “strength”.

Respondent 5: yeah like now (.) look at my bones and my everything you will think I I am a very strong person (.) which a lot of people do (.) but not knowing the inside what is happening to me

The men also reflected on changes in relationships with partner or spouse in the context of masculinity. There were accounts of the physical, practical and emotional support of partners/spouses, accepted ambivalently or appreciation. From an “adjusted masculinity”, acceptance of support was not a relinquishing of male autonomy but was perceived in a relational way.

From an “adjusted masculinity” position, loss of sexual function was tolerable when set against relationality and age identity, as described by Respondent 13:

Respondent 13: (…) since the operation (.) I have become sexually useless (.2) it has harmed me some way (.1) in that part of my life (.) but I am still living and grateful (.) I think that my wife at my age had it happened to me when I was still a young man (.) it would have been a terrible thing (.) because what would I have done to deal with it (.) but as an old man (.) it does not matter to me (.) and my wife is understandable to that situation

There was substantial conversation around work and masculinity, with emphasis on a masculine norm that “a man should work”, and if no longer able to work occupationally to engage in active, practical tasks at home and always try to do “something”. Acceptable or adequate masculinity was largely equated with being occupationally active for most of the men and an active body:

Respondent 11: … I am the kind of a man who does not sit down and do nothing (.) the thing that helps me is not to sit still (…) I do not just sit down and do nothing (.) to me it’s the best of all things and don’t sit back and do nothing

Respondent 11 described how he continued to keep occupationally active with household tasks although he was no longer employed. The men described various adjustments to lifestyle such as no longer using alcohol, not socializing as much as before and change of everyday activities.

Respondent 10: a lot of adjustments (.) as I have said when you were able to to do things (.) and now you cannot (.) or you avoid them.

Although the men described many difficulties, a narrative also appeared of enduring as the “same person” despite the pain, and of adjusting or adapting behaviours around the pain experience. Respondent 5 explained that although he no longer interacted as much as he used to he was still a “sociable person”. Respondent 13 noted that he was “still the same person” although he had to change his ‘duties’ and habits.

Respondent 13: I do not feel less of a man (.) I feel (.) I feel incapable in certain ways like for work and other things (.) but no (.) I do not feel less of a man (.) not not in any way of that sort

The pathway to this “enduring with changes” theme appeared to be an acceptance of the limitations brought about by the pain, the need to modify activities or behaviours and to enlist help when needed. At a level of masculine identity these adjustments were made with an appeal to values and principles such as duty and endurance, and in modifying expectations in relation to hegemonic masculinity, and deeper levels of personal change, as described by Respondent 7:.

Respondent 7: (…) it has changed my life immensely (.) immensely as I say it changed me

At some points, there were accounts of maintaining or forcing an identification with embodied ideals of masculine autonomy, toughness and stoicism (“aligned masculinity”). There were internal and external pressures to conform to cultural ideals. At other points, the men described difficult experiences of change, discomfort and distress, a loss of status, independence and a sense of a diminished, lost or failed masculinity (“yielding masculinity”). There were also moments of an enduring identity despite the pain or an adjusted and changed self and identity along with an acceptance of changes (“adjusted masculinity”).

## Discussion

Following previous work (Blackbeard, 2018), our integration of the narrative account utilized Dialogical Self Theory (DST) with Connell’s Gender Relations Theory (GRT)as a means to conceptualize masculine identity in as embodied dynamic positioning in a personal intra-subjective domain, the interpersonal and intersubjective areas, and social and cultural spaces. Masculine identity was understood as self- and identity-positioning in relation to hegemonic ideals and representations of masculinity in the self-system (Blackbeard, 2018). The individual was both positioned through social and interpersonal influences and was also agentive in enacting identity positions both internally and in relation to others (Blackbeard, 2018). We considered the construction of masculine identities to occur at the interaction of internal and external positions, through which men both internalize and actualize positions in relation to the social and cultural ideals and practices that regulate or legitimate gender relations (Blackbeard, 2018; Hermans, 2015).

### A transforming identity

The men generally did not understate the distress and discomforts of living with chronic pain, some reported variable but continual pain and distress, such as Respondents 1, 8 and 11, where the pain had permeated into deeper levels of identity and personality. Others presented themselves as disconnected from the pain and its effects, with stoicism or having a consistent personal identity, for example, Respondent 5 and 13. Most of the accounts acknowledged losses, constraints, negative emotions and frustrations associated with the pain, sometimes very severe and difficult, but also signalling adjustments and various ways of coping with the pain. The men reported a range of coping strategies; some gained through the Pain Clinic and rehabilitation, such as pain diversions, mental focus, cognitive strategies and pacing and some which might have been gained more through experience, such as asking for help and accepting help, spiritual or religious coping, interaction with other patients and self-responsibility. Some of the strategies were also potentially counterproductive, such as pain denial or dissociation (Respondent 5), attempts to “force” or “control” the pain (Respondent 14) or unregulated use of cannabis. Bernardes et al. (2013) suggest the need for pain physicians to being aware that women learn to accept pain and its associated distress more easily than men. This was possibly true for some of the men in our study, to the extent of acknowledging the pain but minimizing and rationalizing the associated distress. However, there were other men who unreservedly described the pain and associated distress, for some as a state of depression, brokenness and debility and/or others as having required adaptation and endurance. Respondent 1 described his embodied pain as a broken “pillar of life” while at the same time pride of having accepted the changes and support of his wife.

The men described the arrival and persistence of pain as disruptive to their life-world, including the sense of self and identity. They had experienced a wide range of hardships and losses such as a loss of income, status, independence, occupation and influence, and emotional distress, hopelessness and helplessness, a sense of personal failure, stress and worry. At some points, there were stories of enduring difficulties and a strengthened identification with ideal masculinity, to continue being the same person or the same man, even with limitations to enacting and embodying these ideals—in some instances the pain changed “what I do” but not “what I am”. At other points across the narratives, there a sense of living an incomplete or failed masculinity. There were the descriptions of a loss of self-worth, value to others, which was from an internal position (“I as a failed man” or “I as a failing to provide”) or experienced from others (perceptions of “me as an incomplete man” or “me as failed father”) sometimes linked to masculinity in the narratives or for some linked to a loss of personhood, both externally imposed upon the individual through others through expectations and realities of living with chronic pain and internally negotiated through self-positioning and identity adjustments.

The men depicted various times and spaces where an adjusted masculine identity was evidenced in personal experience, such as sitting at home and feeling pressure to be occupationally active (Respondent 11), drive the car or carry the shopping bags (Respondent 5). For some of the men, there was a reiteration of masculine ideals that moved from a literal to a symbolic sense of the enactment or performance of ideals. Instead of being able to demonstrate masculinity through literal hard work and endurance, Respondent 14 suggested that a diligent and enduring approach to life’s challenges maintained a sense of adequate masculinity even when literal income-producing work was no longer possible. No longer in a position to behave sociably as before, Respondent 5 considered himself to still be a “sociable person”. Respondent 13 suggested that although he felt “incapable” in certain areas such as work this did not mean a diminished sense of being a complete man, which for him was more about enduring the difficulties with pride, humour and responsibility. Respondent 13 contrasted how younger version of himself might have struggled more to accept the limitations and losses versus how he had accepted his situation as an older man, where, although not working for income any more, he was still able to exercise masculine obligations through his religious and family duties. Even so, it still “hurt” when he saw his friends going out to work. For others, there was a sense of a permanent loss of role as a provider or breadwinner that could not be replaced or redeemed, experienced with frustration and despondency (Respondent 6 and 7).

Ahlsen et al. (2014) noted that the men in their study sometimes claimed a masculine self that was dissociated from the pain, experiencing disability as a threat to masculine identity which was bridged through reformulating a definition of masculinity that was closer to what was possible in the situation. There was a similar narrative evident from our interviews, with the men reformulating masculine ideals such as control (learning to control the pain—Respondent 14), stoicism and concealing the pain (denying the pain and concealing it from others—Respondent 5), accepting help from others by perceiving this to be unavoidable (accepting there was no choice but to accept help—Respondent 7), still be able to “challenge” a normal man, such as being assertive (Respondent 14).

### Validation and restitution narratives

Across the interviews there was a contrast between the invisibility of the chronic pain and the visible validation provided by medical diagnoses and treatment for chronic pain at the Pain Clinic. This accorded with the findings of Ahlsen et al (2104) who observed that although embracing medical care was a challenge to masculine independence it was outweighed by the visible validation of the chronic pain in a medical context. The treatment experience varied, and although the journey to chronic pain had been very difficult, with doubting treatment providers and miscommunication, they had found a safe haven within a specialized Pain Clinic with support, care and treatment, such as the account by Respondent 6. The experience within the Pain Clinic also appeared to be one of realistic expectations, as it was acknowledged that pain treatments were seldom provided complete or permanent relief, as for example, mentioned by Respondent 7 with regards to spinal blocks. This positive experience was also contrasted with the difficulties perceived to occur in collecting medication at primary care level, where health care workers were perceived to be less understanding and sympathetic, and where medication access and supply appeared to be a problem.

Ahlsen et al. (2014) noted that men developed dynamic resititution narratives in relation to their experiences of chronic pain, sometimes reworked and enriched with the re-telling. Such narratives were influenced by the possibilities and constraints of the present situation of the narrator and generally moved towards future scenarios. For example, the men might develop a narrative of moving from a masculine self that was self-autonomous and in control to a masculine self that received the support of health professionals (Ahlsen et al., 2014). From our findings, it was also evident that some of the men had accepted a new and restored sense of masculine identity with changes to their sense of independence, need for others, acceptance of limitations and finding new meanings in the experiences of living with chronic pain. Accounts converged around chronic pain as an uninvited, life-restricting and unfair experience of suffering and distress for most. The interviews generated a long list of negative emotions associated with chronic pain such as loss of joy, bitterness, hopelessness and frustration. The frustration experienced was in relation to the constraints of the pain conditions, such as activities of daily living (Respondent 7) goals and desires (Respondent 2) and also in significant relationships, such as marital strain and frustrated partners (Respondent 1) or meeting expectations as a family provider (Respondent 5). In some accounts, restitution narratives were developed around the ongoing personal values and characteristics which had continued despite the pain and in some cases even been galvanized by the difficult experiences. In some narratives there was a substitution for the loss of autonomy, in a new sense of gratitude and appreciation, such as Respondent 7’s acceptance that he was no longer self-sufficient but would need to depend on others.

### Embodied changes

Connell and Messerschmidt (2005) have emphasized the close connection of masculinity with men’s bodies and embodiment. Following our theoretical integration of GRT with DST, hegemonic masculinities can be identified with the most valued and influential representations of masculinity within systems of society, social groups, interpersonal domains and within the self-system (Blackbeard, 2018). In discourses of masculinity, a healthy active body is assumed to be necessary in order that ideals of masculinity, such as autonomy, self-efficacy, instrumentality and dominance can be performed (Bernardes & Lima, 2010). Following Bernardes and Lima (2010), men with chronic pain might therefore be positioned as less effective and less able to match a perceived standard for desirable masculinity and have to negotiate new ways to demonstrate masculine acceptability or occupy a space of failed masculinity. Our findings evidenced examples of men dealing with the possibilities of negotiating a new position in the face of embodied challenges and accepting a position of diminished or subordinate masculinity. An embodied ideal of masculine toughness was renegotiated as a mental, moral and emotional resilience in some accounts. For example, Respondent 5 contrasted the perception of the strength others perceived in him for having endured physical adversities with the inner struggles he had endured with a more personal strength, perhaps known only to him or those closest to him. For some, it was important to keep up an appearance of strength and keeping up a standard in the community even if others were not aware of the personal hardships (Respondent 3), to get up, get dressed and carry on living as before as afar as possible (Respondent 14). The observations of Frąckowiak-Sochańska (2021) are relevant here, noting that men’s socialization towards hegemonic practices tends towards a masking of fear and vulnerability or its displacement as anger or impulsive behaviours, which is coupled with an instrumental relationship with the body. The effect of constructions of defended masculinity, such as the ‘aligned masculinity’ position, can be difficulty in adapting to embodied changes and seeking help for psychological distress, which translates into negative health outcomes (Frąckowiak-Sochańska, 2017).

### Limitations of the study

The study was limited by the interview context, and what was both facilitated and constrained in the co-constructive interview space between younger female respondents and the older male interviewees which was therefore conversations across gender, age/generation and in some cases culture and ethnicity. This limitation also offered space for reflexivity and productive dialogue for generating insights and a interpretations.

Secondly, it was also considered that the narratives produced were snapshots of a dynamic process, and that in time the same respondent would develop new versions of their journey with chronic pain in relation to masculinity. The goal of the interview approach (Kvale & Brinkmann, 2015) was to open a space for the respondents to convey their experiences of living with chronic pain in their own words. Following Kvale and Brinkman (2015). The effectiveness of the interviewing approach was partly determined by how sensitively the interviewers engaged with the respondents around the prompting questions. The further factor was extent to which dissent, difference and diversity of opinion could be aired the interview dialogue, as encouraged by Tanggaard (2009). Indications that this had indeed been facilitated were noted in the voicing of reluctance, tension and ambivalence within the interviews, and also in the interviewers taking a tentative approach in reflections following the initial prompts. The training of the interviewing team in a group workshop format was helpful in creating a standardized approach and shared vision for the interviewing process. Credibility was potentially enhanced by the interviewers further engaging with the interview dialogue by transcribing and, for some interviews, translating the interview transcripts from Zulu to English, as conducted in previous research projects (Blackbeard & Lindegger, 2015). There was then the further engagement with the respondents although the respondent validation interviews in which a synopsis of the first interview was prompting of further conversation to validate the themes and co-constructed meanings identified.

Thirdly, there was some difference between the goals of the researchers and the participants’ expectations was the connection of chronic pain experience with masculine identity, and this connection was not what first came to mind for most of these men when they were interviewed about chronic pain. Most of what the men discussed about their experiences of living with chronic pain had no immediate connection to masculinity and it was only with more explicit questions around masculinity that connections could be made beyond description into interpretation.

Fourthly, there was some bias in the selection of the sample to the extent that it recruited men from a Pain Clinic, whereas there might be men outside the clinic who had not accessed Pain Clinic or sought specialized medical treatments for chronic pain or were men who had accessed private sector pain services outside of the public health system. It was also considered that there might well have been many male patients in rural parts of KwaZulu-Natal with chronic pain, such as Zulu men with a background of migrant mining and industry work, who would likely not be receiving pain services beyond the primary health clinic. Many studies have corroborated that avoidance and delayed medical help-seeking is associated with male gender with masculinity norms of self-reliance, avoidance of perceived vulnerability and risk-taking considered as contributing factors (Fleming & Agnew-Brune, 2015). Therefore, the male patients attending the Pain Clinic might not be representative of the clinical population of men with diagnosable chronic pain conditions. There was also a small selection bias, in that participation was invitational and voluntary, and therefore of those invited, two declined to participate and five were not telephonically contactable on the initial call so were not included as the sample reached saturation.

Lastly, Varpio et al. (2017) have noted that qualitative research processes should be applied in a reflexive, nuanced and critical way, with a conceptual basis. The active research process was based in an interpretative phenomenology (life-world interviewing) provided a platform for the researching team’s active engagement in the induction of themes from the material, accounting for researching biases and values through dialogue and team-processes in which divergent interpretations could be discussed. To this end, the process was moderately successful in attaining a goal of credibility, through a multi-layered team approach. The debriefing meetings provided a space for reflecting on the interviewers’ experiences, particularly as young black women interviewing older men, and these reflections could be carried through to the team-based theme identification process.

### Implications for practice, policy and research

Although gender and identity might not be foremost considerations in the delivery of effective pain services for patients with chronic pain, the conclusion of this study is that an inclusive appreciation of patient diversity factors can be helpful in identifying areas where services can be more responsive to the complete needs of patients. Gender-responsive health care in the pain context can provide some validation and relief for distress that is non-discriminatory and inclusive. A sensitive appreciation by the practitioner of the patient’s experience in the context of gender, age and identity can potentially contribute towards effective pain management across the full diversity of patients who require treatment, care and rehabilitation for chronic pain. For example, understanding that older male patients with chronic pain might be negotiating new identity positions can assist the health professional to be empathic of these adjustments and the meanings of pain in relation to age, gender and other social identities. Interdisciplinary teams, standard operating procedures and health system policies for pain management can be similarly informed by the evidence for inclusive recognition of gender, age or other identity towards more effective and accessible services. Qualitative studies around the patient diversity factors and patient experience in context can lay the groundwork for studies that operationalize and systematize the factors which can contribute towards improved patient outcomes in relation to chronic pain. From our study, some of these researchable factors include issues of loss, the meanings of pain in relation to gender, relationships with intimate partners, outward stoicism or denial of distress among male patients, and gender-associated barriers for accessing treatment and rehabilitation. Such research can inform psychosocial and mental health interventions for male patients with chronic pain.

At the level of public health, research such as this study can inform more work around engaging men in medical help-seeking, medical adherence and providing appropriate psychosocial services for men with chronic pain conditions. Public health programmes and policies can be developed which can assist men in managing difficult losses, adjustments and transitions brought about chronic pain, notably in relation to physical trauma from motor vehicle accidents or workplace injuries, and also around what chronic pain conditions can mean for men’s livelihoods, personal identity and indirectly, how this can affect partners and their families.

## Appendix

**Table:** Participant characteristics

Age (years)	Race	Language of Interview	Employment Status	Occupation History	Marital Status	No. of Children
58	Coloured	English	Unemployed	Mechanic	Married	1
65	Black	Zulu	Pensioner	Time clerk	Married	4
44	Black	Zulu	Unemployed	General worker	Single	1
69	Indian	English	Pensioner	Engineering	Married	0
67	Coloured	English	Pensioner	Steelworker	Married	5
52	Black	Zulu	Unemployed	General worker	Cohabiting	4
52	Indian	English	Unemployed	Manufacturing	Married	5
69	Black	Zulu	Pensioner	Machinist	Widowed	3
37	Black	Zulu	Unemployed	Taxi driver	Cohabiting	1
54	White	English	Unemployed	Hotel manager	Divorced	2
66	Black	Zulu	Pensioner	Assistant artisan	Married	9
41	Black	Zulu	Unemployed	Factory worker	Cohabiting	2
71	Indian	English	Pensioner	Property assessor	Married	3
58	Indian	English	Unemployed	Factory worker	Married	3
